# Neuronal Cells Rearrangement During Aging and Neurodegenerative Disease: Metabolism, Oxidative Stress and Organelles Dynamic

**DOI:** 10.3389/fnmol.2019.00132

**Published:** 2019-05-28

**Authors:** Vanessa Castelli, Elisabetta Benedetti, Andrea Antonosante, Mariano Catanesi, Giuseppina Pitari, Rodolfo Ippoliti, Annamaria Cimini, Michele d’Angelo

**Affiliations:** ^1^Department of Life, Health and Environmental Sciences, University of L’Aquila, Abruzzo, Italy; ^2^Sbarro Institute for Cancer Research and Molecular Medicine, Department of Biology, Temple University, Philadelphia, PA, United States

**Keywords:** aging, neurodegeneration, energetic metabolism, mitochondrial dysfunction, oxidative stress

## Abstract

Brain cells normally respond adaptively to oxidative stress or bioenergetic challenges, resulting from ongoing activity in neuronal circuits. During aging and in neurodegenerative disorders, these mechanisms are compromised. In fact, neurons show unique age-related changes in functions and metabolism, resulting in greater susceptibility to insults and disease. Aging affects the nervous system as well as other organs. More precisely, as the nervous system ages, neuron metabolism may change, inducing glucose hypometabolism, impaired transport of critical substrates underlying metabolism, alterations in calcium signaling, and mitochondrial dysfunction. Moreover, in neuronal aging, an accumulation of impaired and aggregated proteins in the cytoplasm and in mitochondria is observed, as the result of oxidative stress: reduced antioxidant defenses and/or increase of reactive oxygen species (ROS). These changes lead to greater vulnerability of neurons in various regions of the brain and increased susceptibility to several diseases. Specifically, the first part of the review article will focus on the major neuronal cells’ rearrangements during aging in response to changes in metabolism and oxidative stress, while the second part will cover the neurodegenerative disease areas in detail.

## Introduction

Aging affects the nervous system as well as other organs. In fact, neuronal cells are subjected to damaged protein accumulation, increased oxidative stress, perturbed energy homeostasis and mutations in nucleic acids. These alterations occur in normal aging and are exacerbated in vulnerable neuronal populations in neurodegenerative diseases.

During aging, the brain gradually declines leading to memory, learning, motor coordination and attention impairment (Alexander et al., 2012; Dykiert et al., 2012; Levin et al., 2014). Elderly people show difficulties in the understanding of rapid speech and complex syntaxes, due to cognitive impairment, but also due to hearing loss (Alexander et al., 2012). Brain decline parallels the aging of other organs, with a progressive deterioration after 50 years (Mendonca et al., 2017). At the 60-year threshold, most people become increasingly inclined to develop neurodegenerative diseases, such as Alzheimer’s disease (AD) and Parkinson’s disease (PD; Mattson, 2004; Kalia and Lang, 2015; Scheltens et al., 2016; Aarsland et al., 2017).

Interestingly, it has been reported that the human brain shrinks in an age-related manner, mostly in the temporal and frontal lobes (Peters, 2006). In fact, the development of brain atrophy during aging may anticipate the risk of developing cognitive impairment and dementia (Jack et al., 2005).

## Aging

### Oxidative Imbalance

**Figure 1:**
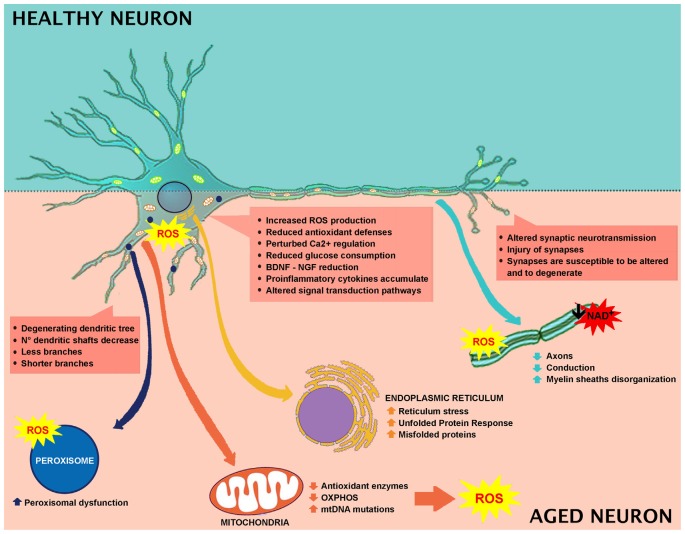
Effect of aging in neuronal cell and involved mechanisms.

Brain is susceptible to oxidative stress because of the elevated content of lipids—especially localized in the neuronal membrane—and the oxidative metabolism occurring in neurons. Tight balance between oxidative stress and the antioxidant system is necessary to preserve the structural integrity and the optimal brain functions (Birben et al., 2012; Castelli et al., 2018). Oxidative damage is involved in aging and age-associated cognitive impairment. During aging, neurons tend to accumulate impaired and aggregated proteins, and damaged mitochondria, as a consequence of oxidative stress. Increase of reactive oxygen species (ROS) production and/or decrease in antioxidant scavengers are the major players in this process (Halliwell, 2001; Figure 1).

Cognitive impairment (Berr et al., 2000; Serrano and Klann, 2004; Kim et al., 2015) and oxidative brain injuries are mainly due to lipid peroxidation products (Calabrese et al., 2004; Richwine et al., 2005; Zhu et al., 2006), protein oxidation (Abd El Mohsen et al., 2005; Rodrigues Siqueira et al., 2005; Poon et al., 2006), and oxidative alterations in nuclear and mitochondrial DNA (mtDNA; Hamilton et al., 2001). Elevated levels of protein carbonylation have been reported in different parts of the brain, among which includes the hippocampus (Abd El Mohsen et al., 2005; Rodrigues Siqueira et al., 2005). The principal neuronal cells ROS source originates from mitochondrial respiration and leads to increased intracellular calcium levels (Halliwell, 2001; Figure 1).

The age-related memory impairment is associated with decreased levels of antioxidants in the brain and plasma (Perkins et al., 1999; Berr et al., 2000; Rinaldi et al., 2003). In physiological conditions, two principal defense mechanisms required to counteract ROS-mediated damage are activated in the brain: the antioxidant enzymes and non-enzymatic antioxidants (Kohen et al., 2000; Kohen and Nyska, 2002). Among the ROS scavenger enzymes, superoxide dismutase (SOD), glutathione reductase, glyoxalase, glutathione peroxidase, and catalase (CAT; Griendling et al., 2000) are comprised. It has been widely demonstrated in various animal models that the intracellular glutathione concentration and the ratio of glutathione/glutathione disulfide (GSH/GSSG ratio) declines in an age-related manner (Sasaki et al., 2001; Sandhu and Kaur, 2002; Rebrin et al., 2003; Wang et al., 2003; Suh et al., 2004, 2005). This event has been described in all of the mammalian brain regions analyzed, including the hippocampus (Calabrese et al., 2004; Balu et al., 2005; Donahue et al., 2006; Zhu et al., 2006). In this condition, Nrf2 (nuclear factor erythroid 2-related factor) transcription factor switches on. The Nrf2 pathway is activated to counteract the accumulation of ROS and reactive nitrogen species since its activation increases the expression of several endogenous antioxidants. Aged animals show a decrease in Nrf2 activation compared to younger subjects (Safdar et al., 2010; Gounder et al., 2012). In agreement, in aged brains, as well as in other tissues, Nrf2 expression is decreased (Duan et al., 2009; Ungvari et al., 2011), paralleled by an increase in inflammatory genes in these organs (Ungvari et al., 2011). For this reason, Nrf2 has been suggested as a potential therapeutic target in diseases with neuroinflammatory and oxidative properties (Salim, 2017).

The mechanisms by which ROS leads to cerebral tissue damage is not clear. ROS may induce the activation of different molecular signaling pathways, including neuroinflammation and neuronal death (Gu et al., 2011; Figure 1), with the involvement of glutamate toxicity, aspartate receptor signaling and glucocorticoid receptor signaling (Albrecht et al., 2010; Nguyen et al., 2011). Biochemically, neurons have different susceptibilities to oxidative stress. For example, amygdala, hippocampus, and cerebellar granule cells are more vulnerable to oxidative stress (Wang and Michaelis, 2010), and accordingly are the first to degenerate. Interestingly, the increased vulnerability of the amygdala and the hippocampus to oxidative stress, together with the lack of antioxidant defenses, alter the biochemical integrity of the brain. Additionally, oxidative stress also affects glutamatergic receptors, responsible for the long-term potentiation and synaptic transmission (Haxaire et al., 2012; Lee et al., 2012; Rai et al., 2013). Altogether, these events underlay oxidative stress-induced cognitive decline.

### Organelle Dynamics

The most typical responses to brain perturbations involve distinct organelles, including the endoplasmic reticulum, which allows the mediation of the Unfolded Protein Response (UPR), the mitochondria, which plays a leading role in regulating apoptosis, the nucleus, in which DNA lesions can trigger stress, and peroxisomes, which have a significant role in the response to oxidative stress (Figure 1).

Alterations in energy or nutrient balance, and changes in calcium signaling or redox status modify endoplasmic reticulum homeostasis, triggering reticulum stress, increased misfolded proteins, and upregulation of UPR. In fact, during aging, there is an imbalance between UPR protective adaptive response and pro-apoptotic signaling in favor of apoptosis (Paz Gavilán et al., 2006; Hussain and Ramaiah, 2007; Naidoo et al., 2008) with the consequent alteration of the capability to maintain proper protein folding and ER homeostasis (Figure 1).

Mitochondrial structure does not change during aging (Ledda et al., 2000; Martinelli et al., 2006). These organelles are mainly localized in neuronal axons and dendrites and are responsible for ATP generation, necessary for maintaining electrochemical neurotransmission and for cell repair (Mattson et al., 2008). One of the most usual consequences of the natural aging process is the reduced level of Ca^2+^-binding proteins.

Beyond their pivotal role in cellular energy metabolism production, mitochondria are also involved in cellular Ca^2+^ homeostasis and nuclear gene transcription regulation (Hou et al., 2012; Yun and Finkel, 2014; Raefsky and Mattson, 2017). Calcium signaling plays a fundamental role in neuronal function, regulation and the structural adaptive mechanism (Cohen et al., 2015). In particular, Ca^2+^ contributes to synaptic activity and is involved in the transmission of the depolarizing signal. During aging the ability of neuronal cells to maintain a proper energy level can be compromised, thus impacting on Ca^2+^ homeostasis and determining weakened control Ca^2+^ dynamics. As a consequence, an aberrant cytoplasmatic Ca^2+^ level occurs, paralleled by perturbed cytoskeletal dynamics and abnormal gene expression (Thibault et al., 2001; Toescu et al., 2004; Gant et al., 2006; Porte et al., 2008). It is clear that altered Ca^2+^ signaling is involved in impaired cognition. The restoration of Ca^2+^ homeostasis in aged rats leads, in fact, to an improvement of cognitive performances (Fukushima et al., 2008; Gant et al., 2015). Moreover, hippocampal neurons are more susceptible to Ca^2+^-excitotoxicity (Camandola and Mattson, 2011). Ca^2+^-dependent calpains activation is involved in neuronal injury and cell death, due to PARP1-mediated apoptosis (Mattson, 2000; Nixon, 2003; Fatokun et al., 2014).

Furthermore, the generation of membrane permeability transition pores is crucial in the apoptosis process (Mattson, 2000). Changes in brain energy metabolism can cause weakening of neuronal functions and alterations leading to neuronal death (Mattson et al., 2008; Grimm and Eckert, 2017; Figure 1).

As indicated above, in the aged brain, a failure in the normal antioxidant defense mechanisms occurs, which renders the brain more vulnerable to the lethal consequences of oxidative stress (Finkel and Holbrook, 2000). It has been reported that mitochondrial-free radicals are responsible for mtDNA damage. Post-mortem brains of elderly showed elevated levels of 8-hydroxy-2-deoxyguanosine in both nuclear DNA (nDNA) and in mtDNA. Moreover, some researchers indicated greater damage in mtDNA than in nDNA in aged rodents (Barja and Herrero, 2000; Barja, 2004; Yang et al., 2008).

During aging, mtDNA oxidative-induced mutations accumulate in post-mitotic tissues, including the brain (Chomyn and Attardi, 2003; Kraytsberg et al., 2003). A growing body of evidence indicates mtDNA mutations as the crucial mechanism leading to aging (Kujoth et al., 2005; Santos et al., 2013; Aon et al., 2016; Scheibye-Knudsen, 2016; DeBalsi et al., 2017; Kauppila et al., 2017; Figure 1). In a recent review, it has been highlighted that accumulation of mutations can induce an alteration in mtDNA replication, leading to electron transport chain activity loss and altered mitochondrial respiration (DeBalsi et al., 2017). In aging, organelle efficiency fails, leading to pathological status (Chauhan et al., 2014). Mitochondrial dynamics are protective in maintaining mitochondria integrity. In fact, during aging, synaptosomal mitochondria shift to a pro-fusion state occurs (Stauch et al., 2014). In the skeletal muscle, the lack of fusion, due to Mfn1 and Mfn2 ablation, induces accumulation of deletions and point mutations in the mitochondrial genome, together with muscle atrophy and mitochondrial alterations (Chen et al., 2010). Moreover, the absence of fusion, resulting from Drp1 ablation in mice adult forebrain, causes mitochondrial dysfunction and altered synapsis transmission, concomitant with decreased ATP production and oxygen consumption (Oettinghaus et al., 2016). The loss of Drp1 affects memory function and synapsis activity, evidencing the crucial role of mitochondrial fusion/fission activity in brain function. Drp1 deletion in post-mitotic Purkinje cells leads to mitochondrial swelling, oxidative damage, accumulation of autophagy markers, leading to neurodegeneration in the cerebellum (Kageyama et al., 2012).

The antioxidant treatment (MitoO and acetylcysteine), in Drp1 KO cells, reduced cell death and mitochondrial swelling (Kageyama et al., 2012). On this premise, efficient mitochondrial dynamics appear to be crucial for maintaining a healthy organelle population for potential therapeutic treatments.

Peroxisomes are essential organelles in eukaryotes and are involved in numerous metabolic pathways and redox homeostasis and contribute to essential brain metabolic processes. Their functional relevance in humans is emphasized by peroxisomal disorders (Trompier et al., 2014). Peroxisomes are implicated in cell aging since they are affected by altered ROS and RNS production (Giordano and Terlecky, 2012; Figure 1). During aging, cells are metabolically less active, and catalase is translocated from peroxisomes to the cytosol. It has been demonstrated that the restoration of peroxisomal catalase levels limits cellular senescence, and therefore, it could be used as a therapeutic target in aging (Giordano and Terlecky, 2012). Furthermore, aging depends on the peroxisome life cycle. After division, both young and old peroxisomes are present, with the latter being damaged by products of peroxisomal metabolism (Beach et al., 2012). The old peroxisomes are thus removed by autophagy to avoid compound accumulation and to maintain organelle homeostasis (Aksam et al., 2007).

### Neuronal Metabolism

During aging, neuronal metabolism is impaired. The human brain has the highest energy requests with respect to any organ system, using more than 20% of the body’s energy, despite comprising only 2% of total body mass (Lin and Rothman, 2014). *In vitro* and *in vivo* studies demonstrated that the brain consumes a large amount of glucose for the maintenance of pre-synaptic and post-synaptic ion gradients for glutamate neurotransmission, and to preserve the resting potential of neurons (Attwell and Iadecola, 2002). Moreover, neurotransmitter signaling needs constant phospholipid remodeling and trans-membrane lipid asymmetries, which represent around 26% of the net energy uptake of the brain (Purdon et al., 2002). In the awake and unstimulated brain, the use of basal energy is already high, and when a stimulus occurs, the energy consumption is even higher.

In the brain, the metabolic fate of glucose varies depending on cell type and on the specific expression of metabolic enzymes. Neurons are mainly oxidative, while astrocytes are generally glycolytic (Ivanov et al., 2014). Neuronal glucose metabolism is multifaceted. It comprises the mechanisms controlling brain glucose consumption, such as the insulin signaling pathways. Brain glucose is transported across the endothelium into astrocytes through GLUT1 (glucose transporter) and transferred into neurons, mostly *via* GLUT3 and GLUT4, participating in the glycolytic pathway. In aged rat brains, decreased glucose uptake and a reduction in neuronal GLUTs have been reported (Yin et al., 2016). During aging, alterations in mitochondria energy-transducing ability, as well as glucose availability occur, together with an impairment in neuronal glucose uptake, increased oxidant production and decreased electron transport chain activity (Yin et al., 2016). A significant role is exerted by insulin and IGF-1 signaling, since they are involved in the regulation and maintenance of cognitive function and brain metabolism (de la Monte and Wands, 2005). Brain aging is closely related to decreased IGF-1 signaling, involving inactivation of the PI3K/Akt pathway (Jiang et al., 2013). Thus, during aging, it is more likely that brain glucose uptake, as well as systemic control of glucose, will fail (Reger et al., 2006). Several preclinical and clinical studies highlight a link between low brain glucose uptake and mild cognitive impairment (Cunnane et al., 2011). In aged brain, glucose hypometabolism and mitochondrial alteration are observed, which are initial indicators of age-related impairment (Small et al., 2000; Mosconi et al., 2008). Moreover, decreased glucose consumption has been described in different brain areas in human subjects with the use of the positron emission tomography analyses of fluorodeoxyglucose uptake (Zuendorf et al., 2003). Rats showed an age-dependent decline in glucose consumption in the hippocampus and cortex, which is linked to impaired cognitive performance (Gage et al., 1984). Synaptic spines are the site of neurotransmission and need a high rate of ATP for maintaining neurotransmitter transporter activities and Na^+^ and Ca^+^ pumps, and for restoring gradients after synapsis activity (Attwell and Laughlin, 2001; Alle et al., 2009; Harris et al., 2012; Rangaraju et al., 2014). During aging, neurons are not able to generate ATP, thus synapses are susceptible to alterations and degeneration (Harris et al., 2012). Numerous factors concur to age-dependent brain hypometabolism. Some clinical studies demonstrated that cerebral blood flow and age are negatively connected (Schultz et al., 1999; Fabiani et al., 2014). During aging, blood-brain barrier (BBB) impairment and brain hypoperfusion lead to decreased import of nutrients, and/or removal of toxins and parenchymal accumulation of blood-derived proteins and immune cells (Zlokovic, 2011; Rosenberg, 2012). Aged mice showed a reduction in white matter, ultrastructural mitochondria modifications and a weak correlation between mitochondria and endoplasmic reticulum (Stahon et al., 2016). Aged brains showed decreased NADH, total NAD and NAD^+^ levels (Bai et al., 2011; Pittelli et al., 2011; Zhu et al., 2015). GLUT1 levels are reduced in an age-dependent manner, in compromised cerebral blood flow, cerebral capillary mass, and glucose uptake, concomitant with augmented BBB leakage (Winkler et al., 2015). These rearrangements precede dendritic spine loss in CA1 hippocampal neurons and are linked to behavioral decline (Winkler et al., 2015).

### Morphological Rearrangement

Wide loss of nerve cells is not present in normal aging, and the regions affected by a neuronal loss are restricted (no more than 10%). The principal age-related neuronal structural alterations involve dendrites reduction in length and number, with a loss of various dendritic spines (Figure 1). Hippocampal DG-CA3 system is involved in regenerative ability, structural plasticity and in the regulation of neurotrophic factors like brain-derived neurotrophic factor (BDNF; Wang and Michaelis, 2010). Oxidative injury of DG-CA could impair remodeling ability, decrease cell proliferation, modify structural plasticity, and alter neurogenesis, collectively impairing normal synaptic neurotransmission. Moreover, dendritic shrinking and amygdala hyperactivity, that could increase synaptic instabilities by altering the hippocampus-amygdala connectivity, have been observed (Kreibich and Blendy, 2004; Brown et al., 2005; Radley et al., 2006; Wood et al., 2010). Synaptic injury is paralleled by axons reduction and disorganized myelin sheaths undergoing segmental demyelination followed by remyelination. These changes contribute to cognitive decline and behavioral impairment that often occurs during normal aging (Peters et al., 2000; Sandell and Peters, 2001, 2003; Peters and Sethares, 2002; Bowley et al., 2010). Myelin is important for insulating axons and guarantees rapid propagation of action potentials, thus alterations in myelin structure induce weak conduction velocity along axons. Additionally, the remyelination process generates shorter myelin internodes (Peters et al., 2000; Sandell and Peters, 2001, 2003; Peters and Sethares, 2002; Bowley et al., 2010). The decrease in conduction velocity along axons, due to changes in myelin sheaths and internodes, renders reaction times longer and interferes with neuronal synchrony, which is implicated in cognitive performance (including attention and memory), as well as sensory and motor functions (Jermakowicz and Casagrande, 2007).

One of the first changes studied in neuronal cells was the accumulation of lipofuscin granules (Moreno-García et al., 2018). Lipofuscin granules, which range between 1 and 3 μm in diameter, appear as brownish particles in neuronal cytoplasm and are probably indigestible residues of lysosomes materials. Since, for many years, lipofuscin was the only one change recognized, its accumulation was an indicative marker of the nervous system aging. Several studies reported that pigmentation of lipofuscin accumulation varies in different regions of the nervous system and that it is accumulated at different rates depending on the brain region. Aggregation of lipofuscin does not strongly affect neuronal metabolism and functional activities (Pannese, 2011).

All the studies on non-human species reported that, during aging, the neuronal loss is restricted to some central nervous system (CNS) areas; however, in human it is still difficult to estimate the percentage of loss but is restricted to specific areas (Merrill et al., 2001; Mohammed and Santer, 2001; Keuker et al., 2004).

**Figure 2:**
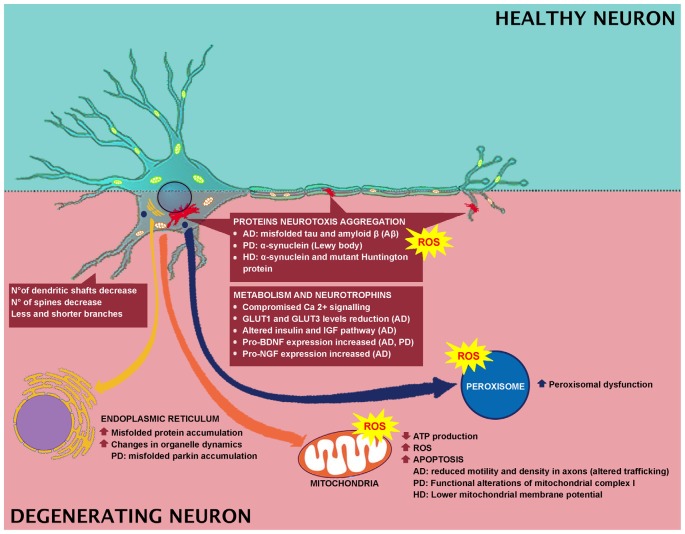
Effect of degeneration in neuronal cell and involved mechanisms.

It has been further reported that aged animals and humans’ axons may present degenerated mitochondria, glycogen inclusions and filaments accumulations (Geoffroy et al., 2016; Salvadores et al., 2017). These modifications trigger axonal impairments. In fact, in aged animals and humans, degenerated or degenerating axons, as well as the reduction in axons number, have been observed (Sandell and Peters, 2001, 2003; Marner et al., 2003; Cepurna et al., 2005; Bowley et al., 2010; Peters et al., 2010). In brain aging and neurodegeneration, the levels of Aβ-peptide and pro-inflammatory cytokines accumulate at an early stage during the pathogenic process (Figures 1, 2). These accumulations trigger the alteration of signal transduction pathways crucial for neuronal health. Neurotrophins signaling is crucial in memory, learning, synaptic function and plasticity, and neuronal cell survival (Smith, 1996; Huang and Reichardt, 2001).

In fact, a preclinical study in aged animals demonstrated that chronic BDNF deficiency induced an age-dependent impairment, thus suggesting that BDNF positively affects brain performance and neuronal survival (Petzold et al., 2015). It has been indicated that during aging, TrkB receptors decrease and that BDNF helps some pituitary function as an intracellular messenger (Rage et al., 2007). BDNF is protective and pro-survival (Castelli et al., 2018), is involved in energy homeostasis control (Xu and Xie, 2016), and is strongly reduced during aging.

Another neurotrophin, Glial cell line-derived neurotrophic factor (GDNF), is altered during aging. GDNF increases in an age-dependent manner in the frontal cortex but not in the hippocampus, suggesting that it exerts its trophic action locally (Matsunaga et al., 2006). Moreover, GDNF is involved in glutamate release and altered glutamate transporters expression, inducing excitotoxicity and triggering neurodegeneration (Farrand et al., 2015).

The glycoprotein nerve growth factor (NGF) is involved in cognitive functions and is strongly decreased during aging. These shreds of evidences suggested that this neurotrophin influences age-dependent cognitive impairment, the maintenance and survival of cholinergic neurons and memory (Yang et al., 2014).

In preclinical studies in transgenic animals, increased BDNF, NGF, and NT3 levels, paralleled by a reduction in number of hippocampal positive amyloid plaques, were reported after insulin-like growth factor 2 (IGF2) treatments (Mellott et al., 2014).

## Neurodegenerative Disease

### Oxidative Imbalance

Neurodegenerative disorders, commonly associated with muscular and cognitive deficits, have pathological hallmarks, including brain atrophy, plaques, neurofibrillary tangles, and aggregates (Kipps et al., 2005; Obeso et al., 2008; Gandhi and Abramov, 2012). In AD, PD and Huntington disease (HD), the main feature is the neurotoxic aggregation of specific proteins in the brain (Figure 2). In specific, AD is characterized by the accumulation of misfolded tau and amyloid β (Aβ) proteins, while in PD and Huntington’s diseases, α-synuclein (α-syn) and mutant Huntington protein (mHtt) accumulate, respectively. Researchers have indicated a link between oxidative stress and development of neuronal plaque, α-synuclein, and mHtt (Li et al., 2013), and a link between the Aβ protein formation and ROS has been reported as well (Behl et al., 1997; Abramov and Duchen, 2005; Shelat et al., 2008). In PD, oxidative stress leads to α-synuclein aggregation in dopaminergic neurons, which in turn induces intracellular ROS formation (Xiang et al., 2013). While in HD, *in vitro* studies indicated that free radicals are implicated in misfolding and accumulation of mHtt-induced neurotoxicity (Pitts et al., 2012). In AD brain, antioxidant enzyme activity is strongly reduced (Ansari and Scheff, 2010) and Aβ-mediated ROS production induces lipid peroxidation, causing reduced membrane permeability and triggering excitotoxicity mechanisms by increased calcium influx (Figure 2). These events lead to altered neurotransmission and cognitive impairment. In fact, ROS has been related to Aβ-induced damage in LTP, resulting in learning and memory impairment, due to altered neuronal transmission (Dumont et al., 2009; Ma et al., 2011; Ma and Klann, 2012; Parajuli et al., 2013). In addition, oxidative stress could be linked to clearance of Aβ. It has been reported that Aβ oxidizes LRP1, thus inducing to the accumulation of the neurotoxic peptide Aβ in the brain. For instance, LRP1 is a multifaceted protein, which controls the efflux of Aβ from the brain to the blood, across the BBB, and LRP1 activity is decreased in AD (Jeynes and Provias, 2008). Therefore, Aβ disrupts this clearance through oxidizing LRP1. LRP1 oxidation is confirmed by the presence of HNE-LRP1 protein adducts in the AD hippocampus (Owen et al., 2010). The altered Aβ clearance triggers Aβ accumulation in the brain, a key factor in AD pathogenesis (Cheignon et al., 2018). Another target for oxidative stress in AD is represented by protein Tau. Indeed, HNE induces alteration in the protein Tau conformation, thus supporting the involvement of the oxidative stress, particularly induced by Aβ, in the AD pathogenesis, by inducing neurofibrillary tangle formation (Liu et al., 2005; Cheignon et al., 2018).

### Organelle Dynamics

In a transgenic model of neurodegenerative diseases, misfolded protein accumulation induces changes in the organelle dynamics, and in the reticulum (Reddy et al., 1999; Rao et al., 2002). UPR impairment has also been described in PD. In fact, with parkin function loss, misfolded parkin substrates accumulate in the endoplasmic reticulum of the dopaminergic neurons of the *substantia nigra*, inducing ER stress and neuronal death (Imai et al., 2001). This event occurs in autosomal recessive juvenile parkinsonism in particular, where mutations in the Parkin gene trigger reticulum stress (Imai et al., 2001). Studies indicated that targeting UPR by inactivation or inhibition represents a potential therapy for neurodegenerative diseases (Brown and Naidoo, 2012).

Mitochondria exert pivotal functions in most neurodegenerative diseases. When mitochondrial dynamics and activities are impaired, low ATP production, high levels of ROS, and apoptosis occur (Suárez-Rivero et al., 2016). Interestingly, mitochondria extracted from lymphoblasts of Huntington’s disease patients show lower mitochondrial membrane potential with respect to the control group (Panov et al., 2002). This finding was also confirmed by mitochondria extracted from the brains of transgenic mice expressing mutant huntingtin protein. The physiological alteration, mitochondrial-mediated oxidative stress and calcium perturbations of the mitochondria induced the onset of behavioral and pathological abnormalities (Mattson et al., 2008). As shown in Figure 2, Ca^2+^ signaling is compromised in neurodegenerative disorders. Interestingly, neurons expressing elevated levels of Ca^2+^–binding proteins are preserved by AD, whereas neurons expressing these proteins at lower levels are subjected to wide impairment. One of the causes of the high vulnerability of AD neurons is the decreased Ca^2+^buffering ability of the neuronal cytosol. Neurons of AD aged patients showed activation of Ca^2+^-dependent proteases (calpain family). Calpain is activated as a response to the increased levels of Ca^2+^ in the cytosol and cleaves various proteins essential for the regular neuronal activity, triggering, and as a consequence, neuronal impairment and apoptosis. PD pathogenesis is attributable to Ca^2+^ dysregulation (Surmeier et al., 2012), therefore, the handling of mitochondrial Ca^2+^ buffering capacity and the pharmacological modulation of L-type channel activity can represent therapeutic strategies for reducing PD progression (Calì et al., 2011, 2014). Functional alterations of mitochondrial complex I are responsible for PD onset (Hu and Wang, 2016). Sporadic PD patients showed reduced complex I in various brain regions, neural and extra-neural tissues (Parker et al., 2008). Complex I deficiency is linked to increased ROS generation (Swerdlow et al., 1996). Moreover, mutations in α-synuclein are typical of PD pathology (Guardia-Laguarta et al., 2014). PD can also be caused by mutations in Parkin and PTEN-induced kinase 1 (PINK1). The Parkin gene is highly expressed in brain tissues, including the *substantia nigra* (Kitada et al., 1998). PINK1 is a mitochondrially-located molecule and has a positive role. A mutation in its kinase domain renders cells susceptible to oxidative stress (Valente et al., 2004).

In AD neuritis, the concomitant depletion of mitochondria or fusion and fission regulators, as well as deficits in axonal transportation and axonopathy, indicate that mitochondrial transport represents one of the causes of impairment (Massano and Bhatia, 2012). Mitochondria Aβ neurons or neurons expressing Amyloid precursor protein exhibit reduced motility and density in axons (Gao et al., 2008; Massano and Bhatia, 2012). Comparably, tau, particularly in its mutant form, alters mitochondrial trafficking in the neuronal cell. This altered trafficking can be reduced by the inhibition of mitochondrial fragmentation (Kausar et al., 2018), suggesting that the impairment of mitochondrial transport is strictly related to mitochondrial fragmentation. This correlation should also be considered in AD, in order to evaluate, in future investigations, if restoring mitochondrial trafficking could prevent Aβ or tau-induced mitochondrial and neuronal impairment. However, it should be explored as to whether alterations of mitochondria in neurodegenerative diseases constitute a primary or a secondary event or are just part of a larger multifactorial pathogenic process.

Numerous evidence indicated that peroxisomes have a crucial role in aging and altered peroxisome functions support the onset of age-related pathologies. In fact, in an *in vitro* study, a connection between peroxisomes and AD has been reported. Primary rat hippocampal neurons were treated with Wy-14.463, a peroxisome proliferator agonist, and it was observed that peroxisomal proliferation protected neurons against Aβ peptide-induced cell death (Santos et al., 2005; Cimini et al., 2009). Moreover, an induction in ABCD3 and ACOX1 expression has been reported in a transgenic AD mouse model that may represent an efficient fatty acid β-oxidation necessary to counteract mitochondrial dysfunctions (Fanelli et al., 2013). Finally, peroxisomal dysfunction induced by thioridazine triggers increased APP and β-secretase expression (Shi et al., 2012). All these pieces of evidence support the relationship between neurodegeneration and peroxisomal dysfunction (Figure 2).

### Neuronal Metabolism

In AD patients, alteration in BBB integrity occurs (Zipser et al., 2007; Zlokovic, 2011). Moreover, the disease-dependent alterations in BBB are faster with respect to normal subjects (Montagne et al., 2015; van de Haar et al., 2016). In addition, an alteration in nutrient and metabolic enzymes has been reported in AD. For instance, GLUT1 and GLUT3 levels are reduced in the AD patients’ brains (Simpson et al., 1994; Harr et al., 1995) and correlate with the reduction of brain glucose consumption and consequent cognitive impairment (Landau et al., 2010; Figure 2).

People with mild cognitive impairment or AD showed a strongly reduced glucose consumption compared to normal aging (Kato et al., 2016). The hypothesis that AD may be considered a type of diabetes mellitus that specifically affects the brain has been recently discussed. In fact, recently, the scientific literature has focused on the fact that AD may be triggered by insulin resistance and insulin deficiency. It has been reported that type 2 diabetes mellitus induces insulin resistance, cognitive impairment, and oxidative stress, mimicking AD neurodegeneration. Moreover, the altered insulin and IGF pathway, as indicated above, represents early and progressive alterations that can trigger the main molecular, histopathological and biochemical lesions of AD patients (Figure 2). Furthermore, it has been reviewed that, as a consequence of the diabetes induction through intracerebral inoculation of streptozotocin, an alkylating antineoplastic agent, animals showed closer features of AD. In addition, common pathophysiological alterations and signaling, such as inflammatory, oxidative stress and PI3K-GSK3β, between AD and diabetes have been indicated. In light of these overlaps, AD has been referred to as type 3 diabetes, since it mimics a kind of diabetes that affects the brain since the AD molecular and cellular characteristics are similar to those observed in type 1 and type 2 diabetes (de la Monte and Wands, 2008; Kandimalla et al., 2017).

### Morphological Rearrangement

In AD there is a substantial loss of neurons but this is not the main factor inducing cognitive impairment (Figure 2). Researchers have explained these phenomena identifying changes in dendrites and axons. For instance, in diseased neurons, the dendritic tree undergoes faster decline, in fact, a decrease in the number of dendritic shafts occurs and the few remaining show fewer and shorter branches, as well as a reduced number of spines (Dickstein et al., 2007; Wang et al., 2018). However, not all the spines are affected in the same manner, since only the thin spines, which are plastic, strongly motile and are involved in learning, are lost (Dumitriu et al., 2010). BDNF is also involved in neurodegeneration (Figure 2) and its strong decrease indicates more vulnerable neuronal populations. This highlights the necessity to detect a potential therapy that targets BDNF signaling (Tapia-Arancibia et al., 2008), since in AD and PD, there is also a concomitant increase in pro-BDNF expression (Budni et al., 2015).

On the other hand, it has been reported that GDNF also has a neuroprotective effect on substantia nigra neurons in PD patients (Gill et al., 2003). In contrast to NGF, an increase in pro-NGF is linked to AD and to mild cognitive impairment. In light of this, NGF, as well as proNGF, could be used as AD targets.

## Conclusion

Aging is a stochastic process dependent on the predictable and random features leading to the accumulation of unrepaired cellular damage, weakened cellular repair, an imbalance in antioxidant defenses and altered metabolism. Healthy aging depends on a combination of lifestyle, individual genetic factors, and external environmental factors. Several reports indicated that glucose hypometabolism, impaired transport of critical substrates, alterations in calcium signaling, mitochondrial dysfunction, and oxidative stress, are mechanisms of aging that render neurons susceptible to degeneration. All of these events trigger morphological rearrangements in neurons, leading to malfunction, compromised transmission, loss of memory and performance (Figure 1).

On this basis, age-related neurodegenerative disorders show a picture far worse with respect to normal aging (Figure 2), characterized by an increase of misfolded proteins, increase of oxidative stress and reduced scavenging capability resulting in massive neuronal loss; mainly in a specific brain area, and leading to heavy alterations in performance, memory and a change in personality.

These findings point towards a multifactorial portrait where different agents, many interdependent, play a significant role during brain aging, and where exacerbation of these factors may expose to the onset of age-related neurodegenerative disease.

## Author Contributions

VC: oxidative stress and general organization of manuscript. EB and MC: neurotrophins. AA and GP: mitochondria. Md’A: energetic metabolism and Figures 1, 2. RI and AC: supervising and editing the manuscript. GP: declare that substantially contributes to the review article in object.

## Conflict of Interest Statement

The authors declare that the research was conducted in the absence of any commercial or financial relationships that could be construed as a potential conflict of interest.
